# Analysis of a Cohort of 165 Pediatric Patients with Human Bocavirus Infection and Comparison between Mono-Infection and Respiratory Co-Infections: A Retrospective Study

**DOI:** 10.3390/pathogens13010055

**Published:** 2024-01-06

**Authors:** Alice Caporizzi, Federica Ravidà, Sara Barneschi, Maria Moriondo, Francesco Nieddu, Silvia Boscia, Mariangela Stinco, Silvia Ricci, Sandra Trapani

**Affiliations:** 1 Postgraduate School of Pediatrics, University of Florence, Meyer Children’s Hospital IRCCS, Viale Pieraccini 24, 50139 Florence, Italy; federica.ravida@unifi.it (F.R.); sara.barneschi@unifi.it (S.B.); 2 Division of Immunology, Meyer Children’s Hospital IRCCS, Viale Pieraccini 24, 50139 Florence, Italy; maria.moriondo@meyer.it (M.M.); francesco.nieddu@meyer.it (F.N.); silvia.boscia@meyer.it (S.B.); silvia.ricci@unifi.it (S.R.); 3 Pediatric Unit, Meyer Children’s Hospital IRCCS, Viale Pieraccini 24, 50139 Florence, Italy; mariangela.stinco@meyer.it (M.S.); sandra.trapani@unifi.it (S.T.); 4 Department of Health Sciences, University of Florence, 50139 Florence, Italy

**Keywords:** human bocavirus, mono-infection, co-infection, children, respiratory tract infection

## Abstract

Introduction: Human Bocavirus (HBoV) is mainly associated with respiratory tract infections. However, its role as respiratory pathogen is not fully understood for a high co-infection rate in symptomatic patients and a significant HBoV detection rate in asymptomatic subjects. This study aimed to describe a large cohort of children with HBoV infection and to compare HBoV mono-infection and co-infections. Methods: We retrospectively reviewed data from 165 children admitted to Meyer Children’s Hospital IRCCS from March 2022 to March 2023 with the diagnosis of HBoV infection, detected using Reverse Transcription qPCR from nasal swabs. Thereafter, we compared patients with HBoV mono-infection (Group A) and those with HBoV co-infections (Group B) in terms of disease severity, established by the length of stay (LOS), the requirement of Pediatric Intensive Care Unit (PICU), and advanced respiratory support (ARS). Results: The median age was 1.5 years; 80% of patients presented with respiratory symptoms. The discharge rate from the emergency department (ED) within 24 h was 42.4%. Most cases (57.6%) were hospitalized, and 7.3% were admitted to PICU due to respiratory failure. Group A comprised 69 patients, and Group B 96 children (95% viral co-infections, 2% bacterial, 3% viral and bacterial). Group A and Group B were similar in hospitalization rate but differed significantly in LOS (median 3 vs. 5 days) and requirement of PICU admission (0 vs. 12 patients, *p* < 0.001). Patients with a respiratory disease history (17.5%) showed significantly longer LOS and more necessity of inhaled bronchodilator therapy. Conclusions: HBoV should be considered a relevant respiratory pathogen especially in viral co-infections. Patients with HBoV co-infections have a higher risk of necessitating advanced respiratory support with more PICU admission and longer LOS; a previous respiratory disease puts them at a higher risk of longer hospitalization.

## 1. Introduction

Human bocavirus (HBoV) is a parvovirus, mainly affecting the lower respiratory and gastrointestinal tracts in childhood all over the world [1,2,3]. It is a small, icosahedral, linear, non-enveloped, single-stranded DNA virus measuring between 18 and 26 nm [4,5,6]. Four strains of the virus have been detected: HBoV1, HBoV2, HBoV3, and HBoV4 [7,8]; the former has been found primarily in samples from the respiratory tract, responsible for upper and lower respiratory tract infections (RTIs) [9]; instead, HBoV 2–4 have been identified mainly in stool, causing gastrointestinal tract infections [10,11]. The transmission is most likely to occur via the respiratory and fecal–oral routes [12]. HBoV infection usually occurs in infants and children aged between 6 and 24 months, being less frequent in children younger than 6 months, maybe due to protection provided by transplacental maternal antibodies, breastfeeding, and less exposure [13,14]; rarely, it can be found in children older than 5 years and adults [15].

Patients acutely infected by HBoV, usually experienced fever and signs or symptoms of lower or upper RTIs, leading to diagnosis of pneumonia, bronchiolitis, bronchitis, rhinitis, tonsillitis, and laryngitis [5,10,16]; gastrointestinal manifestations have been found especially in cases infected by HBoV 2–4. Appetite loss, vomiting, and diarrhea, as well as skin rash, and stomatitis are more rarely described [16,17,18]. Blood exams are usually evocative of viral infection, with C-reactive protein (CRP) and white blood cells (WBC) normal or just slightly elevated [6,18]. Chest radiography frequently shows peribronchial or interstitial infiltrates, hyperinflation, or atelectasis [19].

The HBoV1 virions can infect human airway epithelial cells, inducing airway epithelial damage and inflammation. It has also been demonstrated the genome of HBoV could persist in epithelial cells for months even up to a year after an acute infection [6]. This persistence may explain the detection of viral genomes in about 5–44% of respiratory samples obtained from asymptomatic children [20].

Conversely, many studies on HBoV infection in symptomatic patients highlight the high rate of co-infections, especially with other respiratory viruses, such as Human Rhinovirus (HRV), Adenovirus, and Respiratory Syncytial Virus (RSV) [5]. This multiple detection could be the result of prolonged shedding of HBoV1 in the nasopharynx, for weeks or even months [21,22]. For these reasons, there are still concerns about the role of HBoV, which can be considered a true pathogen or a harmless passenger.

However, in the most recent studies, there is good evidence supporting the hypothesis that HBoV is a genuine pathogenic agent, even when it is the sole infectious agent [6,23,24,25]. Furthermore, viral persistence hinders the diagnosis of acute HBoV infection, making the detection of viral DNA in nasal swabs highly sensitive but little specific. On the other hand, the serology has high specificity but low sensitivity [26]. The presence of mRNA in respiratory secretions is often considered a marker of viral activity and can be used to distinguish acute infection from DNA persistence [27].

To overcome the concern about diagnosis of acute infection, it has been proposed the detection of HBoV in nasopharyngeal swab (HBoV high DNA load on secretion or HBoV mRNA on secretion) and serologic confirmation, with at least one of the following: serum positive IgM, low IgG avidity, or >4-fold IgG titre [6].

This study aimed to evaluate the epidemiology, clinical manifestations, laboratory results, imaging, management, and outcome of a large cohort of children with HBoV infection admitted to a tertiary referral center, over one year. A second aim of the study was to compare data between HBoV mono-infection and HBoV co-infections to discover significant clinical differences.

## 2. Materials and Methods

We conducted a retrospective study on all the children under 16 years admitted to the emergency department (ED), pediatric ward, and pediatric intensive care unit (PICU) at Meyer Children’s Hospital IRCCS (Florence, Italy) from March 2022 to March 2023 with the diagnosis of HBoV infection detected by viral research from nasal swab.

HBoV infection was identified by Reverse Transcription quantitative Polymerase Chain Reaction (RT-qPCR), among an expanded PCR panel testing for the identification of respiratory viruses from nasal swabs. This panel included Influenza A, Influenza B, RSV, Human Metapneumovirus, Human Parainfluenza viruses, Adenovirus, and HRV, in addition to HBoV. To distinguish between high and low viral loads, we considered significant a cycle threshold (ct) below 35, for both HBoV and other pathogens, as previously performed by Silva et al. [28]. Moreover, the presence of SARS-CoV-2 in the samples was evaluated through the antigen level measured with the Lumipulse SARS-CoV-2 Ag kit on the Lumipulse G600II automated immunoassay analyzer (Fujirebio, Inc., Gent, Belgium).

We excluded from our cohort children with HBoV viral load ≥35 ct, and those with other incompatible diagnosis, in whom HBoV has been incidentally detected.

For each patient, all medical data were collected including age, gender, season of admission, necessity for PICU, length of stay (LOS), comorbidities such as prematurity and respiratory disease history, clinical manifestations (as temperature, respiratory or gastrointestinal symptoms, and muco-cutaneous manifestations), and management including drugs and respiratory support. Among laboratory findings, WBC and CRP, when available, were registered and WBC count over 11,000/mcL and CRP more than >1 mg/dL were considered elevated. Radiological data, such as chest X-ray and lung ultrasound (US) were collected, too. Lung US findings were defined as follows: normal air-filled lungs were called pattern A, multiple B-lines, artefacts and micro-consolidation pattern B, and consolidation pattern C.

Based on the number of pathogens in the nasal swab, the selected patients were divided into two groups: Group A, named “HBoV mono-infection”, included children with a high viral load (ct < 35) only for HBoV infection and those with high HBoV viral load (ct < 35) plus another low viral load infection (ct ≥ 35); Group B, named “HBoV co-infection” included children with high viral load (ct < 35) for both HBoV and other identified respiratory viruses. In addition, bacterial co-infections, identified by high ct bacterial PCR from nasal swabs or bronchoaspirates, were included in Group B. Thereafter, we compared these two groups to identify differences in disease severity. As criteria for severity, we relied on the need for hospitalization, LOS, need for PICU, and advanced respiratory support (ARS) which included endotracheal intubation and non-invasive ventilation.

Furthermore, to verify if having comorbidity might lead to a more severe disease, we separately analyzed the subgroups of children with prematurity, defined as birth at <37 weeks of gestational age, and those with respiratory disease history (RDH), including pulmonary bronchodysplasia and/or recurrent lower RTIs.

A specific approval by the local ethical committee was not required because all analysis included in this study had been performed as part of the routine clinical activity. All results have been anonymized.

## 3. Statistical Analysis

Variables were reported as means with standard deviations (SD) or medians with interquartile ranges (IQR) if continuous, and numbers with percentages if categorical. Differences between groups were tested for statistical significance using Wilcoxon rank-sum test for continuous variables and for categorical variables using chi-square test or Fisher’s exact test, as appropriate. All statistical analyses were performed using SPSS statistics 21.0 for Windows.

## 4. Results

### 4.1. Epidemiological Data

**Figure 1:**
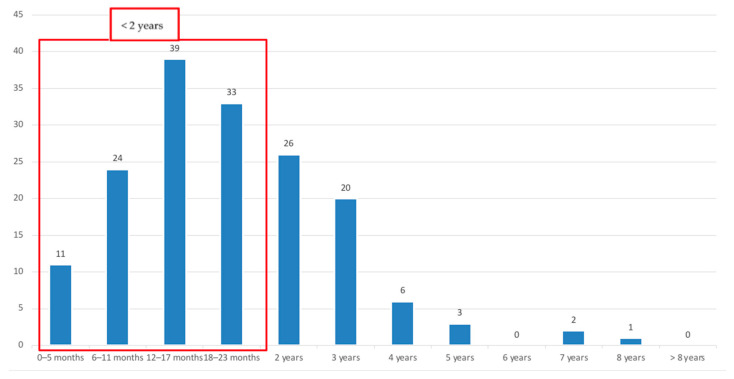
Distribution of HBoV infection by age groups.

Among 296 patients diagnosed with HBoV infection, 165 children were enrolled in our study, having high HBoV viral load (CT < 35). Epidemiological data are shown in Table 1. Male gender was slightly prevalent (*n* = 95; 57.6%). The age range was from 1 month to 8 years, with a median of 1.5 years (IQR 1.1–2.7); 65% of children were under 2 years old (Figure 1). No significant differences in age and gender between Group A and Group B were found.

**Figure 2:**
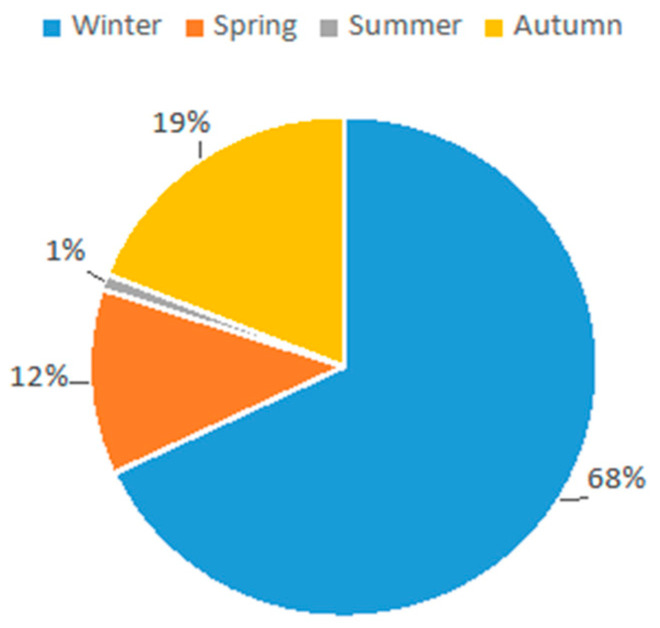
HBoV infections distributed per season of admission in our cohort.

Most acute HBoV infections occurred in winter (*n* = 111; 68%) followed by autumn (*n* = 32; 19%); the incidence dropped during spring (*n* = 20, 12%) and summer (*n* = 2, 1%) (Figure 2).

**Figure 3:**
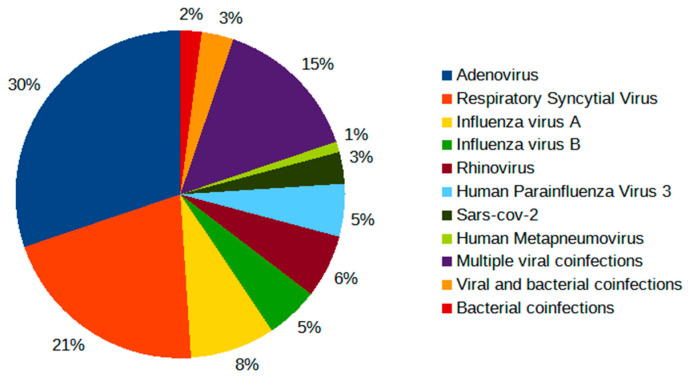
Rates of viral and bacterial co-infections detected in Group B diagnosed with HBoV infection.

Regarding comorbidity, prematurity was detected in five patients (3%), and a previous respiratory disease was found in twenty-nine cases (17.5%). In our cohort, co-infections (*n* = 96; 58.2%) (Group B) were slightly more frequent than mono-infections (*n* = 69; 41.8%) (Group A). Among the co-infections, the viral ones were prevalent (*n* = 91; 95%), especially Adenovirus (*n* = 29, 30%) and RSV (*n* = 20, 21%) (Table 2) (Figure 3).

Bacterial co-infections were rare (*n* = 5; 5%); indeed, only two patients (2%) had a positive bacterial PCR result from bronchoalveolar lavage (BAL), and three cases (3%) had both bacterial and viral positive PCR results from nasal swabs (Table 2).

**Figure 4:**
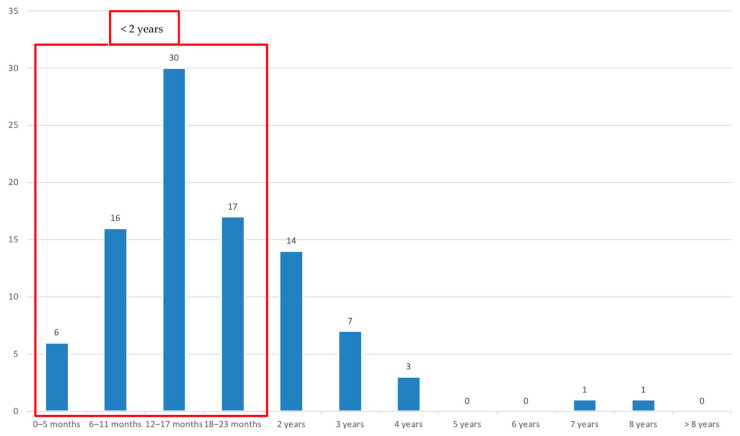
Distribution of hospitalized children by age groups.

Seventy patients (42.4%) were discharged from ED within 24 h, whereas most children (*n* = 95; 57.6%) were hospitalized (Table 1). About the hospitalized children, 73% were under 2 years (Figure 4).

Only 12 patients (7.3%) needed PICU admission for respiratory failure. The hospitalization rate was similar in Group A and Group B, whereas PICU admission was significantly higher in Group B (*p* < 0.001). Among hospitalized patients, the median LOS was 5 days (IQR 2–7); it was significantly longer in Group B, as shown in Table 1. Among children hospitalized in PICU, nine patients (5.4%) required ARS; four were intubated and five were supported with continuous positive airway pressure (C-PAP).

### 4.2. Clinical Manifestations

Most patients (132; 80%) presented mainly with symptoms or signs of RTIs. Most of them (84; 50.9%) had lower RTI features, acting as bronchiolitis, bronchitis, bronchopneumonia, and pneumonia, whereas 48 children (29.1%) experienced symptoms of upper RTIs such as rhinitis, pharyngitis, and laryngitis. Eighteen patients (10.9%) complained of gastrointestinal symptoms (vomiting and diarrhea). Nine patients (5.5%) suffered from febrile seizures. Fever accompanying other manifestations was present in 112 patients (67.8%); 16 children had fever as the unique sign of infection (Table 3). Myositis, stomatitis, and cutaneous rash were occasionally reported. Clinical features were similar in the two groups, except for upper respiratory symptoms, more frequent in Group B (*p* = 0.014).

### 4.3. Laboratory Data

Blood tests were performed for the majority of patients (121, 73.3%), showing only a slight increase of WBC and CRP in 57.8% and 62.8%, respectively. Median values and IQR were, for WBC 12,320 cells/mcL (8900–15,780 cells/mcL) and CRP 1.87 mg/dL (0.56–5.06 mg/dL). The comparison of the inflammatory markers between the two groups A and B did not show any significant difference (Table 3). Blood cultures were performed for six patients and all were negative.

### 4.4. Radiological Findings

Forty-two patients (25.5%) underwent chest X-rays: twenty of Group A and twenty-two of Group B. Findings were similar in the two groups, with the majority showing lung consolidation (Group A: *n* = 15; 75% vs. Group B: 19, 86.4%). Other pathological findings were peri-bronchovascular thickening or interstitial infiltrates in 25% of Group A and 13.6% of Group B, without significant difference. Thirty-three (20%) patients underwent lung US (sixteen of Group A and seventeen of Group B). Pattern A was detected in eight cases (24.3%), equally divided into two groups. The pathological findings were similarly recorded in the two groups. Pattern B was found in seven patients of Group A (43.7%) and four patients of Group B (23.5%). Pattern C was found in five patients of Group A (31.2%) and eight patients of Group B (47%). Just one patient (3%) in Group B had a dysventilated area. The difference in these results was not significant (Table 4).

### 4.5. Management

As shown in Table 5, Group B needed ARS more than Group A (*p* 0.005). Indeed, in Group B, non-invasive ventilation (C-PAP) was performed in five patients (5%) and invasive ventilation with endotracheal intubation in four children (4%). Conversely, none from Group A required ARS. Twenty-four patients (14.5%) were treated with heated humidified high-flow nasal cannula (HHHFNC), in detail, twelve cases (17.4%) belonged to Group A, and twelve (12.5%) to Group B.

Low-flow nasal oxygen therapy was applied to 23 cases (13.9%): 11 (16.9%) of Group A and 12 (12.5%) of Group B. The difference between two groups was no significant. Seventy-three patients (44.2%) needed inhaled short-acting beta-agonists (SABA), thirty-five (50.7%) of Group A and thirty-eight (39.6%) of Group B. Antibiotic therapy was administered to 80 (48.5%) children in both groups (42% in Group A and 53% in Group B) without any statistical difference.

### 4.6. Comorbidities

#### 4.6.1. Prematurity

Overall, in our cohort, five patients (3%) were born preterm: two (2.9%) belonged to Group A, and three (3.1%) to Group B. Among them, one patient from Group A and two from Group B were admitted to the pediatric ward but none required ARS or admission to PICU.

#### 4.6.2. Respiratory Disease History

Twenty-nine patients (17.5%) had a history of recurrent RTIs or pulmonary bronchodysplasia and were named “patients with RDH”. The comparison between patients with such history and those without it (*n* = 136; 84.4%) showed a similar need for PICU admission and ARS. However, the patients with RDH showed a significantly longer LOS (*p =* 0.003, Table 6). Moreover, a higher necessity of inhaled bronchodilator therapy was found in patients with RDH (65.5% vs. 39.7%, *p* = 0.01). To analyze the burden of RDH, we studied Group A and Group B, separately. The results were similar in the two groups, except for LOS, which was significantly higher in patients with RDH belonging to Group B.

## 5. Discussion

HBoV is a parvovirus, isolated about a decade ago, that mainly affects lower respiratory and gastrointestinal tracts in childhood [1,2,3]. Lately, several studies about its clinical manifestations and pathogenetic role have been conducted all around the world, mostly in Asia but also in many European and African countries [13,29]. Overall, the largest cohort of 168 HBoV-infected children was described in Spain, by Pinana et al. [30]. So far, our study conducted on 165 patients can rely on the largest pediatric cohort among the currently available Italian literature [31,32,33]. HBoV1 could appear throughout the year and that depended on climatic and geographical factors; the highest incidence was reported in winter [24,34,35] as occurred in our study with a winter prevalence of 87%.

Earlier studies revealed a slightly higher prevalence of HBoV infection in the male population and children younger than two years [5,10,16,23,24,36], as well as in our patients (57.6%). Indeed, our median age was 1.5 years with a similar epidemiology described by other authors [10,16,23]. Conversely, the incidence of HBoV infection in our population older than 5 years was very low (3.6%), in contrast to the results reported by Wang et al. who found most patients aged over 5 years [5].

Clinical features in our children were mainly represented by respiratory infections, especially lower RTIs (bronchiolitis, pneumonia, and bronchitis), followed by upper RTIs and gastrointestinal involvement, as expected from previous studies [5,10,16,36]. Fever along with other manifestations, was present in most patients (67.8%), as previously reported by Tang et al. [23] and Ji et al. [10]; however, in a minority of cases (9.7%), fever was the unique clinical finding making very difficult the correct diagnosis in these cases.

In most children, HBoV infection had a benign course, in fact, about half (42.4%) of HBoV-infected patients in our cohort did not need to be hospitalized. However, the majority (57.6%) were admitted; this rate was higher in those under two years old (73%); moreover, some life-threatening complications have been described [37,38,39]. Finally, the high detection rate of other viruses in symptomatic patients, in addition to the detection of HBoV also in asymptomatic children, has raised concern about the pathogenicity of HBoV [40,41,42].

In a study from Saudi Arabia, patients with comorbidity needed oxygen more often than healthy children, and they had prolonged LOS [16]. Similarly, in our cohort, longer LOS and a greater need for bronchodilator therapy were required in children with RDH.

In our cohort, prematurity was less prevalent than reported in the Italian general population (3% vs. 7%, according to data presented by the Italian Ministry of Health in 2023 from the “Certificate of attendance at Birth” report) and was not associated with poor prognosis. None of our preterm patients needed either admission in the pediatric ward or PICU or ARS. Nevertheless, prematurity is a well-known risk factor for developing severe and complicated RTIs [43]. Only one patient in our cohort suffered from congenital cardiac disease (restrictive ventricular septal defect). She was an 18-month-old girl, belonging to Group B and developing a respiratory failure, which necessitated PICU admission and ARS.

The disease had a severe course with the requirement of PICU admission for 7.3% of the whole cohort and acute respiratory failure development in nine patients (5.4%), who needed ARS (mechanical ventilation and endotracheal intubation in 2.4% of cases, and C-PAP in 3%). Akturk et al. reported higher rates of PICU admission and mechanical ventilation (33.3% and 20%, respectively) [44]. In our cohort, none developed hepatitis, myocarditis, heart failure, or encephalitis, as complications of HBoV infection previously described [36,37,45] and nobody had a fatal outcome. Our median LOS was 5 days, as according to Alkhaf et al. [16], and slightly shorter than 7 days as reported by several studies [5,23].

Concerning investigations, a slight increase in WBC count and CRP was found in the majority of our patients. Similarly, most children underwent chest X-rays or lung US revealing abnormal findings, consolidation as first, followed by peribronchovascular thickening, and/or interstitial involvement, as according to Zhang et al. [36].

Up to now, there was no clinically approved specific treatment for HBoV infection, and no comparative studies on antiviral drugs have been carried out. As for many viruses, supportive therapy remains the mainstay of treatment for HBoV. This includes providing oxygen for hypoxia, bronchodilators for patients with wheezing, and antipyretics. In our cohort about one-third of children required oxygen support, and 44% bronchodilators.

Regarding multiple infections, a high rate of co-detection is a well-recognized characteristic of HBoV infection [20,21]. The mixed viral infection rate is relatively high in most studies on nasopharyngeal swab samples [15,30,46]. In the largest cohort analyzed by Alkhalf et al., at least 80% of children hospitalized with HBoV infection had one or more co-viral infections, most commonly HRV (45%), adenovirus (30%), and RSV (7%) [16]. Other authors reported similar associations with varying rates; Ji et al. confirmed HRV was the most commonly co-infecting virus (20.5%), followed by RSV (17%) [10]. Our findings were similar to the results reported in the literature; our co-infection rate was 58.2% mainly with viral respiratory pathogens (95%). In particular, the most common association was with Adenovirus (30%), followed by RSV (21%). In contrast, we found a lower prevalence of association with HRV, present only in 6% of patients.

Bacterial co-infections have been described in the literature at different rates, depending on the sample material examined. A dual or triple bacterial co-infection has been detected in 69.6% of nasal swabs in a Chinese cohort [5] (with the *S. pneumoniae* and/or *M. catarrhalis* being the most common) and in 27% of specimens from the lower respiratory tract of the cohort described by Ji et al. (mainly *Escherichia coli* and *Klebsiella pneumoniae*) [10]. In several studies, the most frequent bacterial co-infecting pathogen was *S. pneumoniae* [6,23].

In our cohort, only five patients were detected with bacterial co-infection in nasal swabs or BAL, with a lower bacterial co-infections rate (5%), than previously reported. Such a lower bacterial co-detection rate in our study could be related to the infrequent molecular research for bacteria carried out on nasopharyngeal swabs, sustained by little evidence, in the literature, about the consistency between nasopharyngeal and pulmonary pathogens in children with severe pneumonia [47]. In fact, only a few studies describing this comparison in children have been published yet, mostly related to small cohorts [48]. In a recent study conducted by Wang et al., the concordance between nasopharyngeal swab and BAL sample was largely dependent on bacterial species, with moderate agreement for *M. pneumoniae* and *H. influenzae*, and only poor concordance for S. pneumonia [48].

The comparison between our children with mono-infection and those with multiple infections showed some interesting results. No differences were found in age, clinical manifestations, laboratory findings, imaging, and hospitalization rate in children with HBoV alone vs. those with HBoV co-infections. These findings, in line with those already reported by Petrarca et al. [31], suggested a causative role of HBoV in respiratory and gastrointestinal diseases. Otherwise, our patients with co-infections had a more severe course of the disease, consisting of longer LOS, major risk of acute respiratory failure, and need of ARS. Indeed, all our patients admitted to PICU had multiple infections. Nevertheless, the two groups did not differ in low-flow oxygen therapy, or HHHFNC necessity, or antibiotics. These findings were similar to the results previously reported by Petrarca et al. [31] about LOS, and by Zhang about admission in PICU [36].

Eski et al., found, in Turkey, children with co-infection had higher risks of PICU admission than those with HBoV mono-infection [49]. Similarly, in the study by Pinana et al., children with co-infections required respiratory support for a significantly longer time than those with mono-infections [30]. The presence of multiple respiratory tract co-infections was associated with a severe course and ICU admission also in the Chinese cohort reported by Zhang et al. [36]. In contrast, only in the study reported by Alkhaff et al., patients with co-infections seemed less likely to require an oxygen supply [16].

## 6. Limitations

Our study presented some limitations. HBoV strains were not distinguished by PCR analysis performed by our Hospital Immunology Unit. We did not test HBoV mRNA on secretions and we did not titre serological antibodies of HBoV; this did not ensure the diagnosis of acute infection in our cohort. We studied all co-infections in a single whole group, without comparing different viral associations. Due to the retrospective design of the study, bacterial PCR analysis was not generally performed, but only when deemed appropriate on clinical evaluation; furthermore, only data with positive PCRs were electronically available from our dataset, whereas the total amount of bacterial PCR analysis was not retrievable. Further studies will be necessary to assess the long-term risk for recurrent wheezing in patients with HBoV infection and a better understanding of HBoV interactions with other viruses.

## 7. Conclusions

In conclusion, pediatricians should consider HBoV a true respiratory and gastrointestinal threat, able to cause infectious diseases, even necessitating hospitalization, and even leading to death or life-threatening conditions, such as respiratory failure. Furthermore, our study suggests patients with co-infections have a higher risk to necessitate ARS and PICU admission, as well as a longer LOS. Moreover, including the search for HBoV in nasal swabs in symptomatic patients can lead to the diagnosis of viral infection, avoiding unnecessary antibiotic therapy.

## Figures and Tables

**Table 1:** Epidemiological data, comorbidities, and hospitalization data of HBoV-infected children and comparison between Group A and Group B.

Epidemiological Data	Total (*n* = 165)	Group A (*n* = 69)	Group B (*n* = 96)	*p*-Value
Age (years) < 2, *n* (%)	107 (64.8)	43 (62.3)	64 (66.7)	0.229
2–6, *n* (%)	55 (33.4)	26 (37.7)	29 (30.2)	
>6, *n* (%)	3 (1.8)	0 (0)	3 (3.1)	
Median (IQR), years	1.5 (1.1–2.7)	1.6 (1.1–2.8)	1.5 (1.1–2.7)	0.802
Gender, male, *n* (%)	95 (57.6)	45 (65.2)	50(52.1)	0.09
Comorbidities				
- Prematurity, *n* (%)	5 (3)	2 (2.9)	3 (3.1)	0.933
- RDH, *n* (%)	29 (17.5)	13 (18.8)	16 (16.7)	0.717
Pediatric Ward admission, *n* (%)	95 (57.6)	40 (57.9)	55 (57.3)	0.931
PICU admission, *n* (%)	12 (7.3)	0 (0)	12 (12.5)	<0.001
LOS, days, *n* (IQR)	5 (2–7)	3 (2–6)	5 (2–9)	0.041

RDH: respiratory disease history; PICU: pediatric intensive care unit; LOS: length of stay.

**Table 2:** Co-infections detected in Group B.

Co-Infections, *n* (%)	96 (58.2)
Viral	91 (95)
- Adenovirus, *n* (%)	29 (30)
- Respiratory Syncytial Virus, *n* (%)	20 (21)
- Influenza A, *n* (%)	8 (8)
- Influenza B, *n* (%)	5 (5)
- Human Rhinovirus, *n* (%)	6 (6)
- Human Parainfluenza virus 3, *n* (%)	5 (5)
- SARS-CoV-2, *n* (%)	3 (3)
- Human Metapneumovirus, *n* (%)	1 (1)
- Multiple viral co-infections, *n* (%)	14 (14)
Bacterial	2 (2)
- *S. aureus*, *n* (%) - *H. influenzae*, *S. aureus*, *M. catarrhalis*, *n* (%)	1(1) 1(1)
Bacterial and viral	3 (3)
- *Adenovirus* and *S. Pyogenes*, *n* (%) - *Rhinovirus* and *S. Pneumoniae*, *n* (%) - *Respiratory syncytial virus*, *Adenovirus*, *S. pnemumoniae*, *M. catharralis*, *H. influenzae n* (%)	1(1) 1(1) 1(1)

**Table 3:** Clinical and laboratory data in HBoV-infected children, and in the two groups.

Clinical Manifestations	Total (*n* = 165)	Group A (*n* = 69)	Group B (*n* = 96)	*p*-Value
Upper RTIs, *n* (%)	48 (29.1)	13 (18.8)	35 (36.4)	0.014
Lower RTIs, *n* (%)	84 (50.9)	40 (57.9)	44 (45.8)	0.626
Pneumonia, *n* (%)	18 (11)	9 (13)	9 (9.3)	0.456
Bronchiolitis, *n* (%)	25 (15.1)	8 (11.6)	17 (17.7)	0.28
Gastroenteritis, *n* (%)	18 (10.9)	8 (11.6)	10 (10.4)	0.8
Seizure, *n* (%)	9 (5.5)	3 (4.3)	6 (6.2)	0.596
Fever, *n* (%)	112 (67.8)	42 (60.8)	70 (72.9)	0.102
Fever as unique sign, *n* (%)	16 (9.7)	6 (8.7)	10 (10.4)	0.713
Laboratory data	Total (*n* = 121)	Group A (*n* = 48)	Group B (*n* = 73)	*p*-Value
Altered WBC, *n* (%)	70 (57.8)	29 (60)	41(56)	0.965
WBC (cell/microL), median (IQR)	12,320 (8900–15,780)	12,670 (9162–15,850)	11,500 (8535–15,305)	0.375
Increased CRP, *n* (%)	76 (62.8)	27(56)	49(67)	0.226
CRP (mg/dL), median (IQR)	1.87 (0.56–5.06)	1.5 (0.4–5.0)	1.9 (0.6–5.8)	0.314

RTIs: respiratory tract infections; WBC: white blood cell, IQR: interquartile; CRP: C-reactive protein.

**Table 4:** Radiological findings in children infected by HBoV.

Imaging	Total (*n* = 165)	Group A (*n* = 69)	Group B (*n* = 96)	*p*-Value
Chest X-ray, *n* (%)	42 (25.5)	20 (29)	22 (23)	
Peribronchovascular thickening and/or interstitial infiltrates, *n* (%)	8 (19)	5 (25)	3 (13.6)	0.349
Consolidation, *n* (%)	34 (81)	15 (75)	19 (86.4)	0.349
Lung ultrasound, *n* (%)	33 (20)	16 (23)	17 (17.7)	
Pattern B, *n* (%)	11 (33.3)	7 (43.7)	4 (23.5)	0.129
Pattern C, *n* (%)	13 (39.4)	5 (31.2)	8 (47)	0.129
Dysventilatory area, *n* (%)	1 (3)	0 (0)	1 (5.8)	
Pattern A, *n* (%)	8 (24.3)	4 (25)	4 (23.5)	

**Table 5:** Management of children with HBoV infection divided into the two groups.

Treatment	Total (*n* = 165)	Group A (*n* = 69)	Group B (*n* = 96)	*p*-Value
ARS, *n* (%)	9 (5.4)	0	9 (9.4)	0.005
HHHFNC, *n* (%)	24 (14.6)	12 (17.4)	12 (12.5)	0.536
LFN Oxygen therapy, *n* (%)	23 13.9)	11 (16.9)	12 (12.5)	0.621
Inhaled SABA, *n* (%)	73 (44.2)	35 (50.7)	38 (39.6)	0.155
Antibiotics, *n* (%)	80 (48.5)	29 (42)	51 (53)	0.160

ARS: advanced respiratory support; C-PAP: continuous positive airway pressure; HHHFNC: Heated Humidified High-Flow Nasal Cannula; LFN: low flow nasal; SABA: Short-Acting Beta-Agonist.

**Table 6:** Comparison of disease severity between cases with and without RDH in the whole cohort and in Group A and Group B.

Disease Severity	Total Cases	Cases with RDH	Cases without RDH	*p*-Value
Whole cohort	(*n* = 165)	(*n* = 29)	(*n*= 136)	
Pediatric ward admission, *n* (%)	95 (57.6)	19 (65.5)	76 (55.9)	0.341
LOS, days (IQR)	5 (2–7)	10 (5–12)	4 (2–6)	0.003
PICU admission, *n* (%)	12 (7.3)	3 (10.3)	9 (6.6)	0.483
ARS, *n* (%)	9 (5.4)	3 (10.3)	6 (4.4)	0.201
Group A	(*n* = 69)	(*n* = 13)	(*n* = 56)	
Pediatric ward admission, *n* (%)	40 (57.9)	9 (69.2)	31 (55.4)	0.36
LOS, days (IQR)	3 (2–6)	10 (1.5–12.5)	3 (2–5)	0.092
PICU admission, *n* (%)	0 (0)	0 (0)	0 (0)	
ARS, *n* (%)	0 (0)	0 (0)	0 (0)	
Group B	(*n* = 96)	(*n* = 16)	(*n*= 80)	
Pediatric ward admission, *n* (%)	55 (57.3)	10 (62.5)	45 (56.2)	0.645
LOS, days (IQR)	5 (2–9)	9.5 (5–13)	5 (2–7)	0.017
PICU admission, *n* (%)	12 (12.5)	3 (18-7)	9 (11.2)	0.408
ARS, *n* (%)	9 (9.4)	3 (18.7)	6 (7.5)	0.159

RDH: respiratory disease history; ARS: advanced respiratory support; LOS: length of stay; PICU: pediatric intensive care unit.

## Data Availability

Pseudo-anonymized database will be available upon reasonable request.
